# Anaerobic Microbial Metabolism of Dichloroacetate

**DOI:** 10.1128/mBio.00537-21

**Published:** 2021-04-27

**Authors:** Gao Chen, Nannan Jiang, Manuel I. Villalobos Solis, Fadime Kara Murdoch, Robert Waller Murdoch, Yongchao Xie, Cynthia M. Swift, Robert L. Hettich, Frank E. Löffler

**Affiliations:** a Center for Environmental Biotechnology, University of Tennessee, Knoxville, Tennessee, USA; b Department of Civil and Environmental Engineering, University of Tennessee, Knoxville, Tennessee, USA; c Department of Microbiology, University of Tennessee, Knoxville, Tennessee, USA; d Department of Biosystems Engineering & Soil Science, University of Tennessee, Knoxville, Tennessee, USA; e Bredesen Center for Interdisciplinary Research and Graduate Education, University of Tennessee, Knoxville, Tennessee, USA; f Genome Science and Technology, University of Tennessee, Knoxville, Tennessee, USA; g University of Tennessee and Oak Ridge National Laboratory (UT-ORNL) Joint Institute for Biological Sciences (JIBS), Oak Ridge National Laboratory, Oak Ridge, Tennessee, USA; h Biosciences Division, Oak Ridge National Laboratory, Oak Ridge, Tennessee, USA; University of Massachusetts Amherst

**Keywords:** dichloroacetate, haloacid dehalogenase, fermentation, comparative proteomics, anaerobic catabolic pathways

## Abstract

Dichloroacetate (DCA) commonly occurs in the environment due to natural production and anthropogenic releases, but its fate under anoxic conditions is uncertain. Mixed culture RM comprising “*Candidatus* Dichloromethanomonas elyunquensis” strain RM utilizes DCA as an energy source, and the transient formation of formate, H_2_, and carbon monoxide (CO) was observed during growth. Only about half of the DCA was recovered as acetate, suggesting a fermentative catabolic route rather than a reductive dechlorination pathway. Sequencing of 16S rRNA gene amplicons and 16S rRNA gene-targeted quantitative real-time PCR (qPCR) implicated “*Candidatus* Dichloromethanomonas elyunquensis” strain RM in DCA degradation. An (*S*)-2-haloacid dehalogenase (HAD) encoded on the genome of strain RM was heterologously expressed, and the purified HAD demonstrated the cofactor-independent stoichiometric conversion of DCA to glyoxylate at a rate of 90 ± 4.6 nkat mg^−1^ protein. Differential protein expression analysis identified enzymes catalyzing the conversion of DCA to acetyl coenzyme A (acetyl-CoA) via glyoxylate as well as enzymes of the Wood-Ljungdahl pathway. Glyoxylate carboligase, which catalyzes the condensation of two molecules of glyoxylate to form tartronate semialdehyde, was highly abundant in DCA-grown cells. The physiological, biochemical, and proteogenomic data demonstrate the involvement of an HAD and the Wood-Ljungdahl pathway in the anaerobic fermentation of DCA, which has implications for DCA turnover in natural and engineered environments, as well as the metabolism of the cancer drug DCA by gut microbiota.

## Abstract (precis)

Dichloroacetate (DCA) is ubiquitous in the environment due to natural formation via biological and abiotic chlorination processes and the turnover of chlorinated organic materials (e.g., humic substances). Additional sources include DCA usage as a chemical feedstock and cancer drug and its unintentional formation during drinking water disinfection by chlorination.

## INTRODUCTION

Dichloroacetate (CHCl_2_-COO^–^ [DCA]) is a naturally occurring compound produced through both biological and geochemical processes (1, 2). Marine algae, such as *Asparagopsis* spp., produce DCA, and algal blooms form extensive halogenated dissolved organic matter (chlorine- and iodine-containing metabolites) (3–5). Enzymatic chlorination (e.g., chloroperoxidases) results in substantial chlorination of decaying plant and humic materials leading to the formation of DCA (6–8). Reactive chlorine species (e.g., chlorine radicals generated in photochemical reactions) contribute to organic matter chlorination producing chloroacetates (9, 10). Photochemical degradation of chlorinated hydrocarbons generates DCA, and 1 to 5 μg liter^−1^ DCA has been detected in fog water and rainwater samples (11, 12). Detection of DCA in pristine Antarctic firn is seen as evidence for its natural formation (13, 14). DCA also has various anthropogenic sources, foremost as a consequence of drinking water sanitation. DCA is a common disinfection by-product of water chlorination and occurs broadly in drinking water systems with concentrations reported in the low μg liter^−1^ to hundreds of μg liter^−1^ range (15–17). DCA can also be detected in both bottled and tap water at low μg liter^−1^ levels (18–21). Swimming pool waters treated with chlorine contain DCA, and concentrations reaching 250 μg liter^−1^ have been reported (22). Its use as a therapeutic for a variety of diseases, including cancers and lactic acidosis (23–25), has triggered intense scrutiny by clinical scientists for decades, resulting in rigorous pharmacokinetic, biotransformation, and toxicological studies (26–28). Despite its therapeutic use, DCA is considered a hazardous chemical with cytotoxic and genotoxic effects (29), and it has been classified as an environmental pollutant (30).

In mammalian liver cells, glutathione *S*-transferase (GST) zeta 1 is the primary cytosolic enzyme that transforms DCA to glyoxylate, which is subsequently metabolized via the glyoxylate shunt pathway (31, 32). A novel rho (ρ) class of GST enzymes that catalyze the dehalogenation of DCA to glyoxylate has recently been identified and characterized in the cyanobacterium *Synechocystis* sp. strain PCC 6803 (33). The majority of aerobic bacteria, however, employ distinct enzymes belonging to the group of haloacid dehalogenases (HADs) to convert DCA to glyoxylate via hydrolytic dehalogenation (34–38). Aerobic bacterial degradation of DCA has been studied (39–41); however, the fate of DCA in anoxic environments and anaerobic microbial metabolism of DCA have remained elusive.

A microbial mixed culture, designated culture RM, was derived from pristine freshwater sediment enriched with dichloromethane (CH_2_Cl_2_ [DCM]) as the sole energy source under anoxic conditions (42). Acetate and methane were the final products, and H_2_ was an intermediate during DCM degradation (42–44). Phylogenetic, genomic, and physiological characterization identified the DCM degrader as “*Candidatus* Dichloromethanomonas elyunquensis” strain RM, representing a new genus and species affiliated with the *Peptococcaceae* family (45). Growth of strain RM was strictly dependent on DCM. Other chlorinated solvents, including chloroform, tetrachloroethene, trichloroethene, *cis*-1,2-dichloroethene, 1,1,1-trichloroethane, and 1,1-dichloroethane, did not support growth of strain RM (42, 43). The analysis of the metagenome-assembled genome (MAG) of strain RM identified two putative HADs (46, 47), which triggered the search for additional substrates, specifically chlorinated acetates, that could support growth of strain RM. Here, we report the utilization of DCA as a substrate supporting growth of strain RM, identify a novel HAD that enables the organism to convert DCA to glyoxylate via a glutathione-independent mechanism, and characterize the DCA catabolic pathway. Instead of utilizing DCA as an electron acceptor for reductive dehalogenation (i.e., organohalide respiration), strain RM employs a hydrolytic dechlorination mechanism and ferments DCA to acetate, CO_2_, and H_2_. The new findings advance understanding of the fate of DCA under anoxic conditions and have implications for the flow of carbon and electrons in electron acceptor-depleted environments and the human gut.

## RESULTS

### Dichloroacetate utilization by mixed culture RM.

**FIG 1:**
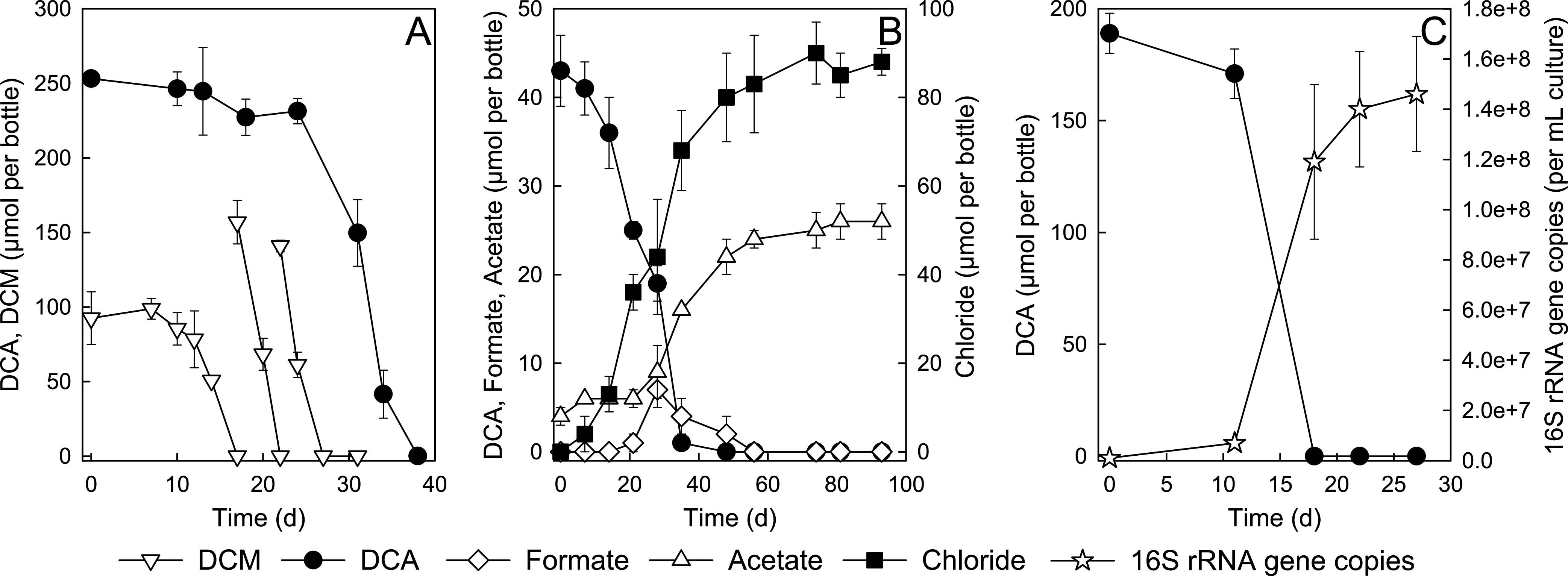
DCA and DCM degradation by mixed culture RM. (A) Utilization of DCA in RM cultures that had consumed an initial feeding of 93 ± 15 μmol of DCM. DCA utilization commenced after a lag phase of about 3 weeks, and consumption was complete within 2 weeks. Replicate DCM-grown cultures rapidly consumed additional DCM feedings without a lag phase. (B) Formation of inorganic chloride, acetate, and formate during DCA catabolism by mixed culture RM. (C) Increase in 16S rRNA gene copies of “*Ca.* Dichloromethanomonas elyunquensis” during DCA catabolism by mixed culture RM under anoxic conditions. The data represent the average from triplicate incubations, and the error bars represent the standard deviations.

When RM cultures that had completely consumed DCM were challenged with 2.5 mM DCA, DCA utilization commenced after a lag phase of about 3 weeks, and consumption was complete within 2 weeks (Fig. 1A). In parallel incubations, DCM-grown cultures rapidly consumed additional DCM (∼150 μmol per bottle) within 1 to 2 days (Fig. 1A). DCA concentrations remained constant in heat-inactivated and no-inoculum controls. Incubations using chloride-free medium demonstrated that the consumption of 43 ± 4 μmol DCA resulted in concomitant formation of 90 ± 7 μmol chloride, indicating that both chlorine substituents were released during DCA catabolism by mixed culture RM (Fig. 1B). During DCA utilization, formate was transiently produced, and up to 7 ± 2 μmol per bottle was observed (Fig. 1B). The terminal product was acetate, and 22 ± 2 μmol—about half the amount of the added DCA (i.e., 43 ± 4 μmol)—was formed (Fig. 1B). In contrast to DCM-grown cultures, DCA-fed cultures did not produce methane. Following the consumption of 2 mM DCA, the cultures were visibly turbid, with optical density at 600 nm (OD_600_) values less than 0.1 optical density unit. Quantitative real-time PCR (qPCR) targeting the 16S rRNA gene of strain RM revealed 141 ± 32-fold increases of cell abundances from (1.04 ± 0.56) × 10^6^ per ml (cells introduced with the inoculum) to (1.46 ± 0.23) × 10^8^ per ml following DCA consumption (Fig. 1C), demonstrating DCA catabolism by strain RM. Monochloroacetate (CH_2_Cl-COO^–^ [MCA]) was never detected in cultures growing with DCA, and MCA could not replace DCA as a growth substrate.

**FIG 2:**
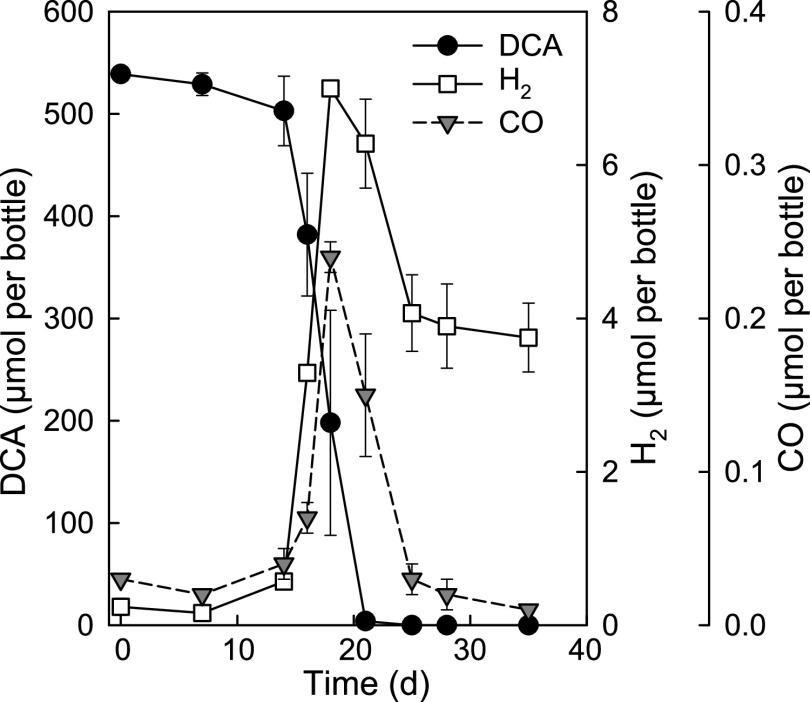
Transient formation of H_2_ and CO during DCA catabolism by mixed culture RM. The data represent the average from triplicate incubations, and the error bars represent the standard deviations.

During growth with DCA (538.8 ± 4.8 μmol of DCA per bottle), H_2_ was intermittently produced, and a maximum amount of 7.00 ± 0.06 μmol of H_2_ was observed (Fig. 2). H_2_ was slowly consumed to a threshold concentration of 1,500 ± 180 ppmv corresponding to 3.75 ± 0.45 μmol H_2_ per bottle (Fig. 2). H_2_ was previously identified as an intermediate of DCM metabolism in culture RM, which supported growth of hydrogenotrophic methanogens (e.g., *Methanospirillum* spp.) and homoacetogens (e.g., *Acetobacterium* spp.) to produce methane and acetate, respectively (43, 44). In addition to H_2_, carbon monoxide (CO) was detected as a transient intermediate during DCA metabolism and increased from 0.03 ± 0.003 μmol to a maximum of 0.24 ± 0.01 μmol per bottle (Fig. 2). The transient formation of H_2_ and CO only occurred in live cultures amended with DCA (see Fig. S1 in the supplemental material). CO formation was not observed in DCM-grown cultures (Fig. S1).

10.1128/mBio.00537-21.1 FIG S1 Change of H_2_ and CO concentrations in abiotic and biotic control incubations and CO concentrations in RM cultures during growth with DCM. Download FIG S1, PDF file, 0.2 MB. Copyright © 2021 Chen et al. 2021 Chen et al. https://creativecommons.org/licenses/by/4.0/ This content is distributed under the terms of the Creative Commons Attribution 4.0 International license.

### Microbial community response to enrichment with DCA.

**FIG 3:**
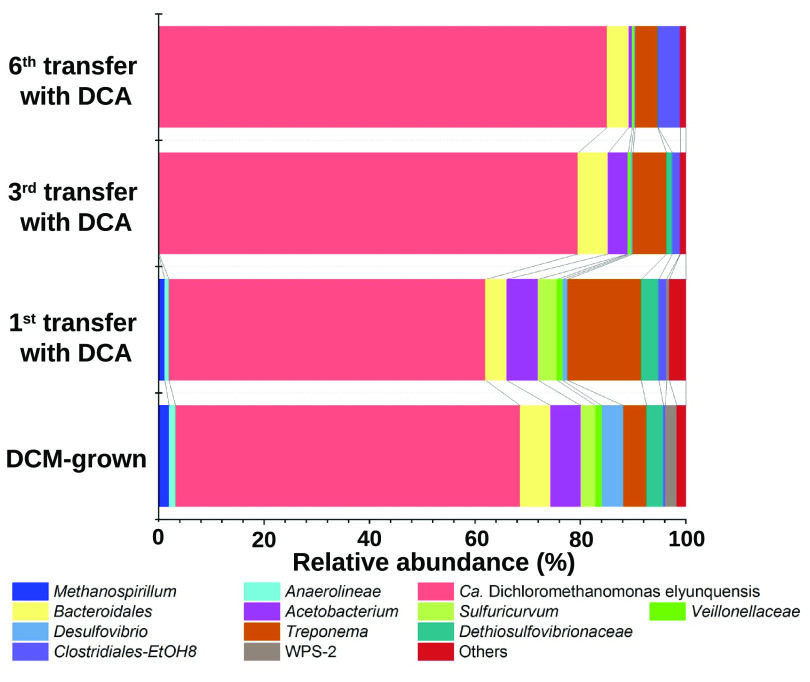
Microbial community structure responses to consecutive transfers of mixed culture RM with DCA as the sole energy source, as revealed by 16S rRNA gene amplicon sequencing. Taxa with relative abundances below 1% were categorized as “Others.” The operational taxonomic units (OTUs) representing bacteria and archaea are reported to the lowest taxonomic rank possible. “*Ca.* Dichloromethanomonas elyunquensis” was the dominant population in mixed culture RM, and continuous transfers with DCA resulted in further enrichment.

16S rRNA gene amplicon sequencing revealed changes in microbial community structure in response to repeated transfers with DCA as the sole energy source. In the first transfer cultures with DCA, strain RM was the dominant population, accounting for approximately 65% of all sequences (Fig. 3; see Table S1 in the supplemental material). Following six consecutive transfers with DCA, the relative sequence abundance of amplicons representing strain RM increased to 87%, implying that strain RM is responsible for DCA degradation (Fig. 3). In DCM-grown cultures, *Methanospirillum* contributed about 2% to the total 16S rRNA gene amplicons (Fig. 3) and was implicated in methane formation (42, 43). Following repeated transfers in mineral salts medium with DCA, *Methanospirillum* sequences were no longer detected (Fig. 3), consistent with the loss of methane formation in cultures grown with DCA. Populations belonging to the genus *Anaerolineae* were also eliminated during repeated transfers with DCA (Fig. 3). The operational taxonomic units (OTUs) representing the genera *Acetobacterium* and *Treponema*, both known to comprise species capable of H_2_/CO_2_ reductive acetogenesis, were maintained at relative abundances of around 1% and 4%, respectively (Fig. 3). OTUs representing *Bacteroidales* were also maintained at a relative abundance of 4 to 5% during consecutive transfers with DCA. The abundances of OTUs representing *Desulfovibrio*, *Dethiosulfovibrionaceae*, *Sulfuricurvum*, and *Veillonellaceae* all declined after repeated transfers with DCA (Fig. 3).

10.1128/mBio.00537-21.8 TABLE S1 Taxonomy, relative abundance, and representative sequences of OTUs identified through 16S rRNA gene amplicon sequencing. Download Table S1, XLSX file, 0.01 MB. Copyright © 2021 Chen et al. 2021 Chen et al. https://creativecommons.org/licenses/by/4.0/ This content is distributed under the terms of the Creative Commons Attribution 4.0 International license.

### Haloacid dehalogenases and DCA dehalogenation.

**FIG 4:**
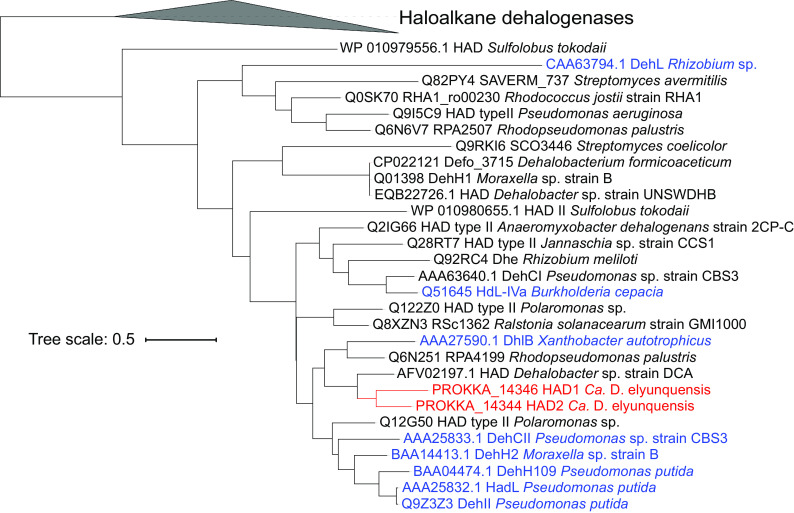
Amino acid sequence-based phylogenetic tree of select HADs. HAD1 (prokka_14346) and HAD2 (prokka_14344) of “*Ca.* Dichloromethanomonas elyunquensis” are shown in red font. Biochemically characterized HADs with demonstrated activity toward DCA are shown in blue font. The scale bar indicates the number of amino acid substitutions per site.

Examination of the genome of strain RM (46) revealed two genes (locus tags prokka_14346 and prokka_14344) encoding putative HADs (EC 3.8.1.2), designated HAD1 and HAD2, respectively. HAD1 and HAD2 shared 60.6% amino acid sequence identity with each other and clustered with biochemically characterized HADs from aerobic bacteria: e.g., Pseudomonas putida, Xanthobacter autotrophicus, *Moraxella* sp., and Burkholderia cepacia (34–36, 38) (Fig. 4).

**FIG 5:**
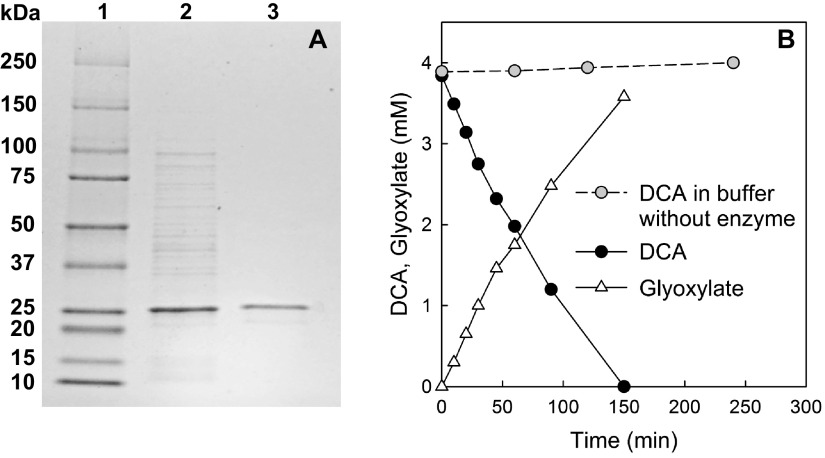
Enzymatic activity of the heterologously expressed HAD1 protein of “*Ca.* Dichloromethanomonas elyunquensis.” (A) SDS-PAGE illustrating HAD1 purification. Lane 1, protein size markers; lane 2, soluble crude extract of E. coli strain FEL153 carrying the *had1* gene (prokka_14346); lane 3, purified His-tagged HAD1 protein. (B) Enzymatic activity of heterologously expressed and purified HAD1 of “*Ca.* Dichloromethanomonas elyunquensis” showing the stoichiometric conversion of DCA to glyoxylate. In abiotic control incubations without protein, DCA was stable. The data shown are from a single experiment, and independent experiments yielded similar results.

To functionally characterize the putative HADs of the strict anaerobe “*Ca.* Dichloromethanomonas elyunquensis” strain RM, the genes encoding HAD1 and HAD2 were cloned and heterologously expressed in Escherichia coli. Assays with cell extracts of the E. coli transformant carrying the *had1* gene revealed the stoichiometric conversion of DCA to glyoxylate (see Fig. S2 in the supplemental material). Extracts of E. coli cells expressing HAD2 did not convert DCA to glyoxylate (Fig. S2). Similarly, cell extracts of an E. coli strain carrying the empty vector without an *had* gene did not catalyze the conversion of DCA to glyoxylate (Fig. S2). Based on these findings, we concluded that HAD1 was responsible for the initial attack on DCA in strain RM. After purification of the His-tagged HAD1 protein using a HisTrap Ni Sepharose column, a single protein band with a size of approximately 25 kDa was observed in SDS-PAGE (Fig. 5A), matching the expected size of the HAD1 protein (i.e., 221 amino acids with a calculated molecular mass of 25.59 kDa). *In vitro* assays demonstrated that the purified HAD1 stoichiometrically converted DCA to glyoxylate at a rate of 90 ± 4.6 nkat mg^−1^ protein (Fig. 5B). The purified HAD1 also converted MCA to glycolate, but at an approximately 70-fold lower rate of 1.3 nkat mg^−1^ protein (see Fig. S3 in the supplemental material). The purified HAD1 protein did not exhibit activity toward DCM, trichloroacetate, and mono- or difluoroacetate.

10.1128/mBio.00537-21.2 FIG S2 Conversion of DCA to glyoxylate in cell-free extracts of E. coli transformants expressing the “*Ca.* Dichloromethanomonas elyunquensis” strain RM *had1* or *had2* gene. Download FIG S2, PDF file, 1.1 MB. Copyright © 2021 Chen et al. 2021 Chen et al. https://creativecommons.org/licenses/by/4.0/ This content is distributed under the terms of the Creative Commons Attribution 4.0 International license.

10.1128/mBio.00537-21.3 FIG S3 *In vitro* conversion of monochloroacetate (MCA) to glycolate using the purified HAD1 protein. Download FIG S3, PDF file, 0.08 MB. Copyright © 2021 Chen et al. 2021 Chen et al. https://creativecommons.org/licenses/by/4.0/ This content is distributed under the terms of the Creative Commons Attribution 4.0 International license.

### Comparative proteome analysis.

**FIG 6:**
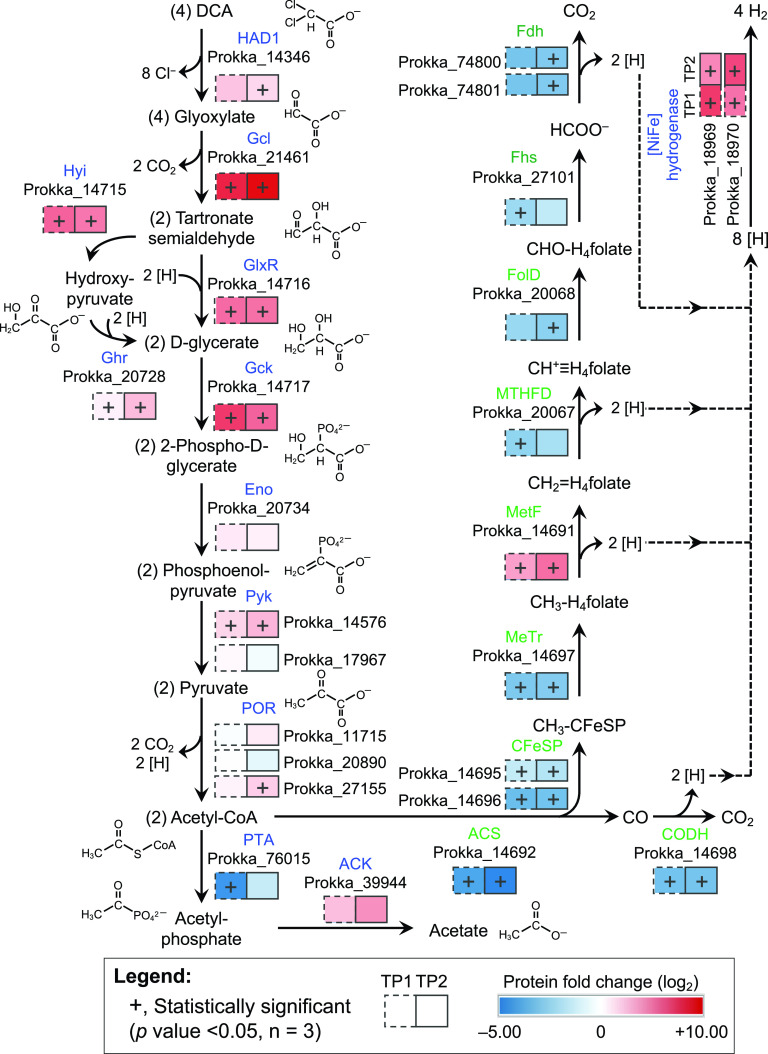
Proposed anaerobic catabolic pathway for DCA in “*Ca.* Dichloromethanomonas elyunquensis” strain RM. The shaded boxes indicate the log_2_ fold change of normalized protein abundance values in DCA- versus DCM-grown cells at TP1 (dashed line boxes) and TP2 (solid line boxes). The protein abundance values represent average from three biological replicate cultures for each growth condition. Boxes marked with “+” signs indicate that the fold changes were statistically significant (*P* < 0.05) in the pairwise comparisons of DCA- versus DCM-grown cells at TP1 and/or TP2. Gene locus tags of each protein are depicted below protein names. Abbreviations: HAD1, haloacid dehalogenase 1; Gcl, glyoxylate carboligase; Hyi, hydroxypyruvate isomerase; Ghr, glyoxylate/hydroxypyruvate reductase; GlxR, 2-hydroxy-3-oxopropionate reductase; Gck, glycerate 2-kinase; Eno, enolase; Pyk, pyruvate kinase; POR, pyruvate-flavodoxin oxidoreductase; PTA, phosphate acetyltransferase; ACK, acetate kinase; ACS/CODH, acetyl coenzyme A synthase/carbon monoxide dehydrogenase; CFeSP, corrinoid iron-sulfur protein; MeTr, methyltransferase; MetF, methylene-tetrahydrofolate (H_4_folate) reductase; MTHFD, methylene-H_4_folate dehydrogenase; FolD, formyl-H_4_folate cyclohydrolase; Fhs, formyl-H_4_folate synthase; Fdh, formate dehydrogenase. WLP proteins are depicted in green font, and proteins involved in DCA reduction to acetate are shown in blue font. The fold change values are shown in Table S2.

The 3-week adaptation time required for DCM-grown cultures to commence DCA utilization (Fig. 1A) suggested that proteins involved in metabolizing DCA are inducible. A comparative global proteomic analysis between DCA- and DCM-grown cells was performed to elucidate differential abundance expression of proteins involved in DCA versus DCM metabolism in strain RM. A complete list of proteins identified under the different growth conditions at two sampling time points (i.e., immediately before the 2nd electron donor amendment [TP1] and near the end of electron donor consumption [TP2]; see Fig. S4 in the supplemental material) is presented in Table S2 in the supplemental material. HAD1 (prokka_14346) was detected in the proteomes of DCA- and DCM-grown cells; however, the expression was greater in DCA-grown cells at both time points, with log_2_ fold changes of +2.38 and +1.69. HAD2 (prokka_14344) was not detected in cultures grown with either substrate (Fig. 6), an observation consistent with the *in vitro* enzyme activity results (Fig. S2) and indicating that HAD2 is not involved in DCA metabolism.

10.1128/mBio.00537-21.4 FIG S4 Growth of “*Ca.* Dichloromethanomonas elyunquensis” during DCA and DCM catabolism and collection of samples for comparative proteome analysis. Download FIG S4, PDF file, 2.1 MB. Copyright © 2021 Chen et al. 2021 Chen et al. https://creativecommons.org/licenses/by/4.0/ This content is distributed under the terms of the Creative Commons Attribution 4.0 International license.

10.1128/mBio.00537-21.9 TABLE S2 Summary of proteins identified in RM cultures grown with DCA or DCM at two sampling points. Samples were collected immediately before the 2nd electron donor amendment (TP1) and near the end of electron donor consumption (TP2); approximately 95% of the initial amount of electron donor was consumed (Fig. S4). Imputed values are shown in orange. Download Table S2, XLSX file, 0.6 MB. Copyright © 2021 Chen et al. 2021 Chen et al. https://creativecommons.org/licenses/by/4.0/ This content is distributed under the terms of the Creative Commons Attribution 4.0 International license.

A glyoxylate carboligase (Gcl; prokka_21461), which catalyzes the decarboxylation of glyoxylate and the ligation to a second molecule of glyoxylate to form the three-carbon compound tartronate semialdehyde, was among the most highly abundant proteins in DCA-grown cells. The log_2_ fold changes in Gcl in DCA- versus DCM-grown cells were +8.09 and +9.26 at the TP1 and TP2 time points, respectively, and both log_2_ fold changes were above the statistically significant level (*P* < 0.05 [Fig. 6]). A series of enzymes involved in the stepwise conversion of tartronate semialdehyde to acetyl coenzyme A (acetyl-CoA), including 2-hydroxy-3-oxopropionate reductase (GlxR; prokka_14716), hydroxypyruvate isomerase (Hyi; prokka_14715), glyoxylate/hydroxypyruvate reductase (Ghr; prokka_20728), glycerate 2-kinase (Gck; prokka_14717), and pyruvate kinase (Pyk, prokka_14576), were significantly more abundant in DCA-grown cells at both time points (Fig. 6). Other predicted pathway enzymes, such as enolase (Eno; prokka_20734), a second Pyk (prokka_17967), and pyruvate-flavodoxin oxidoreductase (POR; prokka_27155, _20890, and _11715), did not show statistically significant abundance fold changes in DCA-grown versus DCM-grown cells (Fig. 6); however, they were all detected in the proteome, with potential roles in the transformation of 2-phospho-d-glycerate to acetyl-CoA. The presence of phosphate acetyltransferase (PTA; prokka_76015) and acetate kinase (ACK; prokka_39944) enzymes, both of which are encoded on the genome, was confirmed in the analyses of both DCA- and DCM-grown cells. Interestingly, PTA was less abundant in DCA-grown cells, while the ACK abundance was higher than that in DCM-grown cells. Although more information is required to understand the regulatory controls of these enzymes, their detection in cultures growing with DCA suggests an acetyl transfer reaction followed by a dephosphorylation reaction to convert acetyl-CoA to acetate, which was measured as a terminal product in DCA-grown cultures (Fig. 6). In addition, all of the Wood-Ljungdahl pathway (WLP) proteins encoded on the genome of strain RM were detected in the proteome of DCA-grown cells, suggesting the involvement of WLP enzymes in DCA catabolism (Fig. 6).

## DISCUSSION

Previous studies focused on DCA catabolism in aerobes; however, the fate of DCA under anoxic conditions remained obscure. The anaerobic mixed culture RM could be maintained with DCA as the sole source of energy, and the molecular analyses implicated the DCM-degrading bacterium “*Ca.* Dichloromethanomonas elyunquensis” strain RM in DCA catabolism. The findings demonstrate that specialized anaerobes metabolize DCA and illustrate that the range of substrates strain RM can utilize is not limited to DCM.

### Initial enzymatic attack on DCA.

Both chlorine substituents were released during DCA degradation, and acetate was an end product (Fig. 1B). The stepwise reductive dechlorination of trichloroacetate (CCl_3_-COO^–^ [TCA]), DCA, and MCA via reductive dechlorination is thermodynamically favorable, with Gibbs free energy changes of –171.2, –154.0, and –152.0 kJ per reaction, respectively, under standard conditions with H_2_ as electron donor (48). The transformation of TCA to DCA has been observed when TCA was incubated with mouse or rat gut microflora under anoxic conditions (49). Reductive dechlorination of TCA to DCA was explicitly demonstrated in an axenic culture of Geobacter thiogenes (formerly Trichlorobacter thiogenes) strain K1; however, a cryptic sulfur-sulfide redox cycle was involved in dechlorination, and the organism apparently does not perform organohalide respiration (50, 51). The genome of strain RM encodes three putative reductive dehalogenases (RDases), and two of them were expressed during growth with DCM (47). One of these RDases (prokka_14638) was detected during growth with DCA, albeit at very low abundance. The experimental efforts did not generate any evidence for reductive dechlorination. MCA, the product of a single reductive dechlorination (hydrogenolysis) reaction, was neither detected as an intermediate nor supported growth of mixed culture RM. Furthermore, the utilization of DCA as an electron acceptor in organohalide respiration should result in the formation of stoichiometric amounts of acetate; however, only about 50% of the initial amount of DCA was recovered as acetate in RM cultures. The experimental data indicate that strain RM ferments DCA to acetate, H_2_, and CO_2_ and does not utilize DCA as an electron acceptor in organohalide respiration (Fig. 6).

The integrated physiologic, proteogenomic, and enzymatic studies pinpoint a novel HAD involved in converting DCA to glyoxylate in a strictly anaerobic bacterium. HADs belong to a large superfamily of hydrolases with diverse substrate specificities and catalyze the hydrolytic dehalogenation of 2-haloalkanoic acid to the corresponding 2-hydroxyalkanoic acids (38, 52). Many aerobic bacteria, including members of the genera *Pseudomonas*, *Xanthobacter*, and *Moraxella*, possess HADs that convert DCA to glyoxylate to initiate DCA metabolism and growth (34–36, 38). HADs are not sensitive to O_2_ and do not require any cofactors such as O_2_ or glutathione for activity. In contrast, mammalian liver cells employ glutathione *S*-transferases (GSTs) to convert DCA to glyoxylate (31–33). GSTs play central roles for detoxification of various groups of harmful compounds, such as halogenated nitrobenzenes, arene oxides, and quinones (53). DCM dehalogenases of aerobic and facultative aerobic methylotrophic bacteria, which catalyze the conversion of DCM to formaldehyde, also belong to GSTs (54, 55). The active site of GSTs is the thiol group of the glutathione cofactor, which in its reduced form performs a nucleophilic attack on nonpolar compounds containing an electrophilic carbon, nitrogen, or sulfur atom (53). GSTs strictly require glutathione as a cofactor and have been found in eukaryotes, some aerobic and facultative aerobic methylotrophic bacteria, and recently in a cyanobacterium (33), but never in strict anaerobes (53). HADs, in contrast, employ the carboxyl group of an aspartate residue in the active center to carry out an SN_2_ nucleophilic attack on the α-carbon atom of the halogenated carboxylate substrate to displace a halogen atom and produce an enzyme-bound ester intermediate. The ester bond is subsequently hydrolyzed to produce the corresponding d-2-hydroxyalkanoate and regenerate the aspartate residue (56, 57). Although both HADs (EC 3.8.1.2) and GSTs (EC 2.5.1.18) are capable of removing chlorine substituents from DCA, yielding the same products (i.e., glyoxylate and inorganic chloride), their reaction mechanisms are fundamentally different and belong to distinct enzyme classes.

### DCA fermentation pathway.

Based on 16S rRNA gene sequence analysis, “*Ca.* Dichloromethanomonas elyunquensis” strain RM is phylogenetically related to Syntrophobotulus glycolicus (45), an isolate capable of fermenting glyoxylate to glycolate, CO_2_, and H_2_ (58, 59). Based on physiological and enzymatic evidence, *S. glycolicus* strain FlGlyR^T^ (DSM 8271) was proposed to metabolize glyoxylate via malyl-CoA to glycolate, CO_2_, and H_2_ under anoxic conditions without an external electron acceptor (58). Genes encoding HADs were not found on the genome of *S. glycolicus* (60), and strain FlGlyR^T^ could not utilize DCA as a growth substrate. The HAD-catalyzed DCA dehalogenation leads to the formation of glyoxylate, which is subsequently fermented by strain RM, generating acetate, CO_2_, and H_2_. In addition to DCA, strain RM is also able to ferment glyoxylate (see Fig. S5 in the supplemental material), consistent with the observation that glyoxylate is an intermediate of DCA metabolism. In contrast to *S. glycolicus*, strain RM does not possess the canonical genes for malyl-CoA lyase and malate dehydrogenase, and we never detected glycolate in culture supernatant. Instead, the comparative proteome analysis revealed high expression of glyoxylate carboligase (Gcl; prokka_21461) in DCA-grown cells (Fig. 6). This enzyme catalyzes the condensation of two molecules of glyoxylate to form tartronate semialdehyde, suggesting this C_3_ compound is a pathway intermediate. The comparative proteome analysis further revealed the abundance of proteins (i.e., GlxR, Hyi, Ghr, Gck, Eno, Pyk, and POR) potentially involved in converting tartronate semialdehyde to acetyl-CoA via glycerate and pyruvate (Fig. 6). Glyoxylate metabolism through tartronate semialdehyde and the glycerate pathway was proposed previously in the oxalate-degrading anaerobic bacterium Oxalobacter formigenes based on the detection of enzymatic activities in the cell-free crude extract, specifically the activities of glyoxylate carboligase (Gcl), tartronic semialdehyde reductase (GlxR), and glycerate kinase (Gck) (61).

10.1128/mBio.00537-21.5 FIG S5 Growth of “*Ca.* Dichloromethanomonas elyunquensis” in mixed culture RM with glyoxylate as the sole electron donor as determined by 16S rRNA gene-targeted qPCR. Download FIG S5, PDF file, 0.2 MB. Copyright © 2021 Chen et al. 2021 Chen et al. https://creativecommons.org/licenses/by/4.0/ This content is distributed under the terms of the Creative Commons Attribution 4.0 International license.

The genome of strain RM encodes a complete WLP, and the corresponding proteins were detected in DCA-grown cells, indicating the involvement of the WLP in DCA metabolism. Based on gene content, the tricarboxylic acid (TCA) cycle is incomplete, and the oxidation of acetyl-CoA through the TCA cycle is not possible. Half of the acetyl-CoA formed during DCA metabolism is likely oxidized to CO_2_ via the reverse WLP generating reducing equivalents (i.e., electrons and protons) (Fig. 6). The WLP has also been implicated in glyoxylate metabolism in the thermophilic homoacetogenic bacterium *Moorella* sp. strain HUC22-1, which ferments glyoxylate to acetate and CO_2_ via malyl-CoA rather than tartronate semialdehyde and glycerate (62). Anaerobic DCM metabolism also proceeds via the WLP (43, 63), and all WLP proteins were highly expressed in strain RM cells during growth with DCM (47). To facilitate direct comparisons, the pathway postulated for anaerobic DCM metabolism in strain RM is shown in Fig. S6 in the supplemental material. The detection of formate and CO as intermediates during DCA metabolism (Fig. 1 and 2) lends further support for the involvement of the WLP in DCA metabolism. CO is an obligatory intermediate of the WLP, generated by the bifunctional enzyme CO dehydrogenase/acetyl-CoA synthase (CODH/ACS) during the reduction of CO_2_ (64). CO was not detected in cultures grown with DCM, which may be explained by the direction of the CODH/ACS reaction during DCA versus DCM metabolism, *viz.*, the oxidative direction during DCA degradation versus the reductive direction during DCM catabolism. Another possible explanation is the mineralization of DCM to CO_2_ and H_2_ via the oxidative WLP, with a small fraction of the DCM carbon being assimilated through anabolic reactions (i.e., via the reductive route of the WLP) with CO as an intermediate (Fig. S6). Although strain RM metabolizes both DCM and DCA via the WLP, the proteomic data indicate relative higher expression levels of WLP proteins (with the exception of MetF) in DCM-grown cells.

10.1128/mBio.00537-21.6 FIG S6 Postulated anaerobic DCM metabolic pathway in “*Ca*. Dichloromethanomonas elyunquensis” strain RM. DCM is metabolized via the Wood-Ljungdahl pathway (WLP). Download FIG S6, PDF file, 0.04 MB. Copyright © 2021 Chen et al. 2021 Chen et al. https://creativecommons.org/licenses/by/4.0/ This content is distributed under the terms of the Creative Commons Attribution 4.0 International license.

H_2_ was detected as an intermediate during DCA degradation in culture RM, similar to what has been observed in *S. glycolicus* cultures fermenting glyoxylate. The genes encoding two putative group 4 H_2_-evolving [NiFe]-hydrogenases (prokka_18969 and prokka_18970) are present on the genome of strain RM, and both [NiFe]-hydrogenases were highly expressed during growth with DCA (Fig. 6). Very likely, one or both [NiFe]-hydrogenases is involved in H_2_ formation during DCA metabolism by catalyzing the reduction of protons generated from the oxidation of acetyl-CoA.

Degradation of both DCA and DCM generated H_2_, but methanogenesis only occurred in DCM-grown cultures. Repeated transfers with DCA eliminated methanogens (Fig. 3), hinting at possible toxic effects of DCA on methanogens (65). H_2_ consumption in DCA-grown mixed culture RM was attributed to bacteria performing H_2_/CO_2_ reductive acetogenesis, e.g., *Acetobacterium* and *Treponema* (66, 67). H_2_ was eventually scavenged to 1,500 ± 180 ppmv during growth on DCA (Fig. 2), which is consistent with the H_2_ consumption threshold concentration range reported for H_2_/CO_2_ reductive acetogenesis as the terminal electron accepting process (68, 69).

“*Ca.* Dichloromethanomonas elyunquensis” strain RM has resisted isolation—presumably due to the requirement for a hydrogenotrophic partner population to remove H_2_ (Fig. 3). Elevated H_2_ partial pressures inhibit DCM (43) and DCA degradation (see Fig. S7 in the supplemental material) indicative of strict syntrophy, and strain RM relies on H_2_-scavenging populations to metabolize DCM and DCA. Based on the physiological observations and proteomic data, DCA metabolism in strain RM generates acetate, CO_2_, H_2_, chloride (Cl^–^), and biomass (Fig. 1) and proceeds according to equation 1:

(1) $${\text{4 CHCl}}_{\text{2}}{\text{COO}}^{–}+{\text{ 6 H}}_{\text{2}}\text{O}\to {\text{CH}}_{\text{3}}{\text{COO}}^{–}+{\text{ 8 Cl}}^{–}+{\text{ 5 H}}^{+}+{\text{ 6 CO}}_{\text{2}}+{\text{ 4 H}}_{2}\;\Delta G{{}^{\circ}}^{\prime }=-225{\text{ kJ (mol DCA)}}^{-1}$$

The generated H_2_ is consumed in H_2_/CO_2_ reductive acetogenesis leading to acetate formation according to equation 2:

(2) $${\text{4 H}}_{\text{2}}+{\text{ 2 CO}}_{\text{2}}\to {\text{CH}}_{\text{3}}{\text{COO}}^{–}+{\text{ H}}^{+}+{\text{ 2 H}}_{\text{2}}\text{O }\;\Delta G{{}^{\circ}}^{\prime }=-23.75{\text{ kJ (mol H}}_{2}{\text{)}}^{-1}$$

Therefore, DCA catabolism in mixed culture RM proceeds according to equation 3, with half of the DCA being reduced to acetate and the other half being oxidized to CO_2_, which is consistent with the experimentally measured stoichiometry, *viz.*, the ratio of DCA degraded versus acetate generated was 2.15 ± 0.05 to 1 (Fig. 1B). Based on these observations, half of the acetate formed is directly derived from DCA, and the other half is generated via reductive acetogenesis by utilizing H_2_ generated during DCA catabolism:

(3) $${\text{4 CHCl}}_{\text{2}}{\text{COO}}^{–}+{\text{ 4 H}}_{\text{2}}\text{O}\to {\text{2 CH}}_{\text{3}}{\text{COO}}^{–}+{\text{ 8 Cl}}^{–}+{\text{ 6 H}}^{+}+{\text{ 4 CO}}_{2}\;\Delta G{{}^{\circ}}^{\prime }=-249{\text{ kJ (mol DCA)}}^{-1}$$

10.1128/mBio.00537-21.7 FIG S7 Inhibitory effect of H_2_ on DCA degradation in mixed culture RM. Download FIG S7, PDF file, 0.9 MB. Copyright © 2021 Chen et al. 2021 Chen et al. https://creativecommons.org/licenses/by/4.0/ This content is distributed under the terms of the Creative Commons Attribution 4.0 International license.

### Implications.

Natural and anthropogenic processes introduce DCA into the environment, and the prevalence of *had* genes in the genomes of aerobic bacteria can be viewed as a consequence of ubiquitously present DCA (6–8, 21). DCA formation has been reported in various terrestrial environments, as well as marine and peat bog ecosystems (7, 8). Information about DCA pool sizes is only available from coniferous forest soils, which contain approximately 300 ng g^−1^ soil (6). Based on this information, we calculate that the global DCA amount in coniferous forest soils alone exceeds 8 × 10^9^ kg (assuming an area of 40 × 10^6^ km^2^, a surface soil depth [A horizon] of 40 cm, and a soil bulk density of 1.6 g/cm^3^) (70). Because information about DCA fluxes in environmental systems is lacking, DCA turnover may be substantial even in the absence of measurable DCA pools. The heretofore unrecognized anaerobic DCA degradation pathway via glyoxylate likely constitutes the dominant route of DCA catabolism under electron acceptor-depleted conditions, with implications for carbon and electron flow in anoxic environments. DCA fermentation generates acetate and H_2_, both of which are central intermediates during carbon cycling and can fuel anaerobic food webs. Therefore, the abiotic and biotic formation and subsequent fermentation of DCA may be relevant processes for sustaining microbial activity in energy-depleted environments such as the deep subsurface. The findings also have bearing on the clinical use of DCA as a drug and future studies should explore if members of the gut microbiota have the ability to ferment DCA and assess the responses of the gut microbiome to DCA treatment (71, 72).

## MATERIALS AND METHODS

### Chemicals.

DCA (purity, >99.8%) and DCM (>99.95%) were purchased from Sigma-Aldrich Co. (St. Louis, MO) and Acros Organics (Fair Lawn, NJ), respectively. Gas mixtures with H_2_ partial pressures of 10, 50, and 100 ppmv were purchased from Airgas (Radnor, PA), and CO gas (>99.0%) was purchased from Sigma-Aldrich Co. and used for standard curve preparation. All other chemicals used were analytical reagent grade or higher.

### Microorganisms and cultivation.

Mixed culture RM was derived from pristine freshwater sediment and maintained with DCM as the sole energy source for 8 years (42, 45). Culture RM was routinely grown in 160-ml glass serum bottles containing 100 ml of anoxic, bicarbonate-buffered (30 mM, pH 7.3) basal salts medium reduced with 0.2 mM sulfide and 0.2 mM l-cysteine (73). The vessels were sealed with black butyl rubber stoppers (Bellco Glass, Inc., Vineland, NJ) under a headspace of N_2_/CO_2_ (80/20 [vol/vol]), with 5 to 10 μl neat DCM (78 to 156 μmol) provided as the sole electron donor prior to inoculation from a DCM-grown culture (5% [vol/vol]). Cultures that had consumed the initial dose of DCM received 1 to 2 mM DCA to examine its potential utilization as an energy source. Following the consumption of DCA, RM cultures were repeatedly transferred (3% [vol/vol]) with DCA as the sole energy source before the experiments reported herein were initiated. All culture vessels were incubated at 30°C in the dark without agitation. To quantitatively measure inorganic chloride release during DCA degradation, incubations were conducted in chloride-free medium with bromide salts substituting for chloride salts.

Syntrophobotulus glycolicus strain FlGlyR^T^ (DSM 8271) was purchased from DSMZ-German Collection of Microorganisms and Cell Cultures GmbH (Braunschweig, Germany) and was grown in the anoxic, bicarbonate-buffered (30 mM, pH 7.3) basal salts medium as described above, with glyoxylate (5 mM) as the sole energy source. To test DCA as a potential substrate for *S. glycolicus* strain FlGlyR^T^, 2 and 5 mM DCA replaced glyoxylate in medium inoculated from a glyoxylate-grown *S. glycolicus* culture (3% [vol/vol]).

### DNA extraction and quantitative real-time PCR.

For DNA extraction, 5 ml of culture suspension was periodically collected during a growth cycle on DCA or DCM and filtered onto 0.22-μm-pore Durapore membranes (Millipore, Cork, Ireland). DNA was extracted using the DNeasy PowerSoil DNA isolation kit (Qiagen, Hilden, Germany) following the manufacturer’s protocol. 16S rRNA gene-targeted qPCR was used to monitor growth of strain RM in cultures grown with DCA or DCM. qPCR assays used primers and a probe specifically targeting the 16S rRNA gene of strain RM (45) and were conducted using an ABI ViiA7 real-time PCR system (43, 45).

### 16S rRNA gene amplicon sequencing.

To monitor the microbial community response to repeated transfers with DCA, 16S rRNA gene-based amplicon sequencing targeting the V4 region of both bacterial and archaeal 16S rRNA genes was performed following established procedures (74, 75). The amplicons were sequenced on the Illumina MiSeq platform (San Diego, CA), and data analysis was performed with the QIIME v.1.9.1 software package (76). Raw sequencing reads were jointly paired, demultiplexed, and trimmed to a length of 250 bp, and chimeric reads were removed. After quality control, over 100,000 individual sequences were obtained for each library generated with DNA samples collected from consecutive transfers with DCM or DCA. Operational taxonomic units (OTUs) were picked via the default UCLUST pipeline (77) and filtered at a 0.005% threshold. Taxonomic assignments were performed using the RDP classifier trained against the Greengenes 16S rRNA gene database (version 13.8) (78). Taxonomy, relative abundance, and sequences of representative OTUs are shown in Table S1. The most abundant sequence within each taxon was chosen as the representative sequence for each OTU.

### Heterologous *had* gene expression, purification, and *in vitro* activity testing.

The pET-28a(+) expression vector backbone was used to clone and express *had* genes carrying an N-terminal His tag. “*Ca.* Dichloromethanomonas elyunquensis” *had* genes (locus tags prokka_14344 and prokka_14346) were amplified from DNA extracted from DCA-grown cells using Phusion Flash High-Fidelity PCR master mix (Thermo Fisher) and primer sets NJ762 (5′-CTAGAAATAATTTTGTTTAACTTTAAGAAGGAGATATACCATGATTAGAGCTGTAGTCTTTGATGCC-3′) and NJ763 (5′-AGCAGCCGGATCTCAGTGGTGGTGGTGGTGGTGCTCGAGTTCAAATATTCTTAGTTTTGAGGGCCAAC-3′) and NJ764 (5′-CTAGAAATAATTTTGTTTAACTTTAAGAAGGAGATATACCATGATTAAGGCATGCGCATTTGATG-3′) and NJ765 (5′-AGCAGCCGGATCTCAGTGGTGGTGGTGGTGGTGCTCGAGTTTATAGGCCTTTAACTTTTTGAGCC-3′), respectively. PCR products were cleaned using the UltraClean PCR Clean-Up kit (MO BIO Laboratories, Inc., or Zymo DNA Clean and Concentrator). The pET-28a(+) vector backbone was digested with the restriction endonucleases BamHI, NcoI, NdeI, and NotI for untagged constructs and BamHI, NdeI, and NotI for tagged constructs and gel extracted to remove any remaining supercoiled plasmid. The linearized vector and *had* gene inserts were then cotransformed into electrocompetent E. coli strain BW25113 cells with preinduced λ Red recombinase from plasmid pKD46 (79) to allow for homologous recombination. All PCR amplicons in recombinant vectors were sequence verified using Sanger sequencing. Following sequence verification, recombinant vectors pNJ100 (carrying prokka_14344) and pNJ101 (carrying prokka_14346) were introduced into E. coli strain BL21(DE3) (New England BioLabs) for overexpression and purification. The supplemental material provides additional information about primers (Table S3A), plasmids (Table S3B), and E. coli strains (Table S3C) used for the heterologous expression of *had* genes.

10.1128/mBio.00537-21.10 TABLE S3 Primers, plasmids, and E. coli strains used for cloning and overexpression of *had* genes. Download Table S3, XLSX file, 0.01 MB. Copyright © 2021 Chen et al. 2021 Chen et al. https://creativecommons.org/licenses/by/4.0/ This content is distributed under the terms of the Creative Commons Attribution 4.0 International license.

E. coli strain BL21(DE3) carrying an *had* expression plasmid was grown in 300 ml of Terrific Broth (Thermo Fisher) with 50 μg ml^−1^ kanamycin at 37°C and 150 rpm to an optical density at 600 nm (OD_600_) of 1.0. Cultures were then induced with 1 mM isopropyl β-d-1-thiogalactopyranoside (IPTG; Thermo Fisher) and incubated overnight at room temperature at 150 rpm. Cells were collected by centrifugation at 9,000 × *g* for 25 min, suspended in 25 mM Tris-HCl buffer (pH 7.5), and sonicated at 50% amplitude (Branson Sonifier 250; Branson Sonifiers, Danbury, CT) in an ice bath for 8 min with a 50% duty cycle (30 s on and 30 s off). The lysate was centrifuged at 38,000 × *g* for 20 min, and the supernatant was passed through a 5-ml HisTrap Ni Sepharose column (GE Healthcare) using an ÄKTA Prime fast protein liquid chromatography (FPLC) system (GE Healthcare, Pittsburgh, PA). Proteins were eluted with 25 mM Tris-HCl buffer (pH 7.5) containing 100 mM NaCl and 300 mM imidazole. Fractions containing protein were combined, and the elution buffer was exchanged with 4 ml of 100 mM Tris-HCl (pH 8.0) by using a 10-kDa-cutoff Amicon Ultra-4 filter unit (Millipore). Protein concentrations were quantified using the Bradford assay (80), and protein purity was examined by SDS-PAGE and Coomassie blue staining. A standard protein marker (Bio-Rad, Hercules, CA) with protein sizes ranging from 10 to 250 kDa allowed size estimations. The purified HAD1 protein stock solutions (0.6 mg ml^−1^) were frozen immediately and stored at −80°C. The N-terminal hexahistidine tag was retained for all experiments.

Enzyme assays were conducted in 100 mM Tris-HCl buffer (pH 8.0) in Eppendorf tubes in a total assay volume of 0.5 ml. DCA was added at a concentration of 4 mM, and the reactions were started by adding 3 μg of HAD protein. The tubes were agitated with 150 rpm at 30°C. Aliquots of 50 μl were collected over time, acidified with 1 μl of 1 M H_2_SO_4_ to quench the reaction, centrifuged at 17,000 × *g* for 5 min at room temperature, and analyzed by high-performance liquid chromatography (HPLC) to determine the concentrations of DCA and glyoxylate. HAD enzyme activity was calculated based on the formation of glyoxylate: 1 nkat is the amount of enzyme that generates 1 nmol of glyoxylate per second.

### Global proteomics of RM cultures grown with DCA versus DCM.

Prior to proteomic analysis, cultures were consecutively passaged at least three times on the same substrate (i.e., DCA or DCM). Following the consumption of 361.0 ± 19.5 μmol of DCA and 373.2 ± 5.8 μmol of DCM, the respective cultures received one additional feeding of the respective substrate. Samples for proteomic analysis were collected upon the consumption of the first (time point 1 [TP1]) and the second (TP2) substrate feedings (Fig. S4). Cells grown with DCA or DCM were collected from triplicate cultures by passing 100 ml of culture suspension through Sterivex 0.22-μm-pore filter units (EMD Millipore Corporation, Billerica, MA). The outlet of a filter unit was capped, and 1.5 ml of boiling SDS lysis buffer (4% SDS [wt/wt] in 100 mM Tris-HCl buffer, pH 8.0) was added. Following gentle agitation on a shaker with three-dimensional (3D) gyratory action for 1 h at room temperature, a 3-ml plastic syringe was connected to the cartridge’s inlet, the unit was inverted, and as much lysate as possible was transferred into the syringe. The filter units were rinsed once with 500 μl of fresh lysis buffer at room temperature, and the recovered volumes were combined. The cell lysates were then subjected to trichloroacetic acid precipitation followed by urea denaturation, reduction, blocking of disulfide bonds, and tryptic digestion (trypsin/protein ratio of 1:50 [wt/wt]) as described previously (81). The protein contents in crude and peptide extracts were quantified using the bicinchoninic acid (BCA) assay (Pierce Biotechnology, Waltham, MA). Peptide extracts were stored at −80°C until liquid chromatography-tandem mass spectrometry (LC-MS/MS) analysis. Proteomics data sets from culture RM were obtained with an Orbitrap Q Exactive Plus mass spectrometer (Thermo Fisher Scientific, Waltham, MA) equipped with an electrospray ionization (ESI) source and interfaced with a Proxeon EASY-nLC 1200 system. Peptides (2 μg) from each sample were suspended in solvent A (2% acetonitrile, 98% water, 0.1% formic acid) and injected onto a 75-μm-inner-diameter microcapillary column packed with 35 cm of Kinetex C_18_ resin (1.7 μm, 100 Å; Phenomenex). Peptides were separated using a 90-min gradient from 2% to 30% solvent B (80% acetonitrile, 20% water, 0.1% formic acid), followed by an increase to 40% solvent B within 10 min and a 10-min wash with 98% solvent A. The flow rate was kept at 250 nl min^−1^. MS data were acquired with the Thermo Xcalibur software version 4.27.19, with a topN method, where N was capped to 15. Other scanning and spectral data collection parameters were similar to those reported previously (82). All spectral data collected in this study have been deposited in the MASSIVE and ProteomeXchange repositories with identifiers MSV000086520 and PXD022742, respectively (ftp://massive.ucsd.edu/MSV000086520/).

### Peptide and protein identification by database searching.

MS/MS raw data files from culture RM were searched against a database of sequences annotated from the draft genome of “*Ca.* Dichloromethanomonas elyunquensis” (accession no. LNDB00000000), to which common contaminant proteins were appended (www.thegpm.org/crap). The MyriMatch v2.2 algorithm was used for standard database searching and was set to the same parameters described previously (82, 83). Confidently identified peptides at a false-discovery rate below 1% were assembled into proteins using the IDPicker v.3.1 software (84). Every protein in the data set was identified with at least two unique peptide sequences. For label-free quantification, the MS1-level peptide precursor intensities were extracted from IDPicker with IDPQuantify, summed by protein, and then divided by the sequence length of the protein to which they matched (85). Protein abundance values were log_2_ transformed and then normalized by mean central tendency analysis with Inferno RDN (https://omics.pnl.gov/software/infernordn). The Perseus software (86) was then used to filter proteins with non-zero abundance values in two out of three biological replicates in at least one growth condition and time point. After data filtering, undetected proteins (i.e., proteins with missing abundance values) were imputed with a simulated Gaussian distribution of low-abundance values to provide non-zero abundance metrics at the detection threshold. This approach enables statistical analyses across the entire data set, as is commonly done in proteome measurements (87, 88). Pairwise *t* test comparisons were conducted to identify proteins having statistically significant abundance changes (*P* < 0.05) between growth conditions at each respective substrate feeding time point.

### Analytical methods.

DCA, glyoxylate, formate, and acetate were analyzed on an Agilent 1200 series HPLC system equipped with an Aminex HPX-87H column (Bio-Rad, Hercules, CA) operated at 30°C and a multiple-wavelength detector set to 210 nm. Operation was isocratic using 4 mM H_2_SO_4_ as the eluent at a flow rate of 0.6 ml min^−1^. Aqueous samples (200 μl) were acidified with 4 μl 1 M H_2_SO_4_ and filtered prior to HPLC analysis. The identification of peaks was based on retention times of authentic standards, and quantification was achieved using external calibration curves. DCM was measured by manually injecting 0.1-ml headspace samples into an Agilent 7890A gas chromatograph (Santa Clara, CA) equipped with a DB-624 column (60-m length, 0.32-mm inside diameter, 1.8-μm film thickness) and a flame ionization detector as described previously (89). Chloride ions were measured with an ion chromatograph using a Dionex ICS-2100 system equipped with a 4- by 250-mm IonPac AS18 hydroxide-selective anion-exchange column (Thermo Fisher Scientific, Waltham, MA) operated at 30°C. The 10 mM KOH eluent was delivered at a flow rate of 1 ml min^−1^, and an ERS 500 suppressor (4 mm) was set at a current of 57 mA. To follow the formation of H_2_ and CO, 0.5 ml of culture headspace samples was injected into a Peak Performer 1 gas chromatograph coupled with a reducing compound photometer (Peak Laboratories, Mountain View, CA) with detection limits for H_2_ and CO below 8 ppb by volume (ppbv).
