# Supplementary material for: Anaerobic Microbial Metabolism of Dichloroacetate
Source: mBio. 2021 Apr 27;12(2):e00537-21. doi: 10.1128/mBio.00537-21 (PMC8092247; doi:10.1128/mBio.00537-21)
Supplement: FIG S1 [file mBio.00537-21-sf001.pdf]

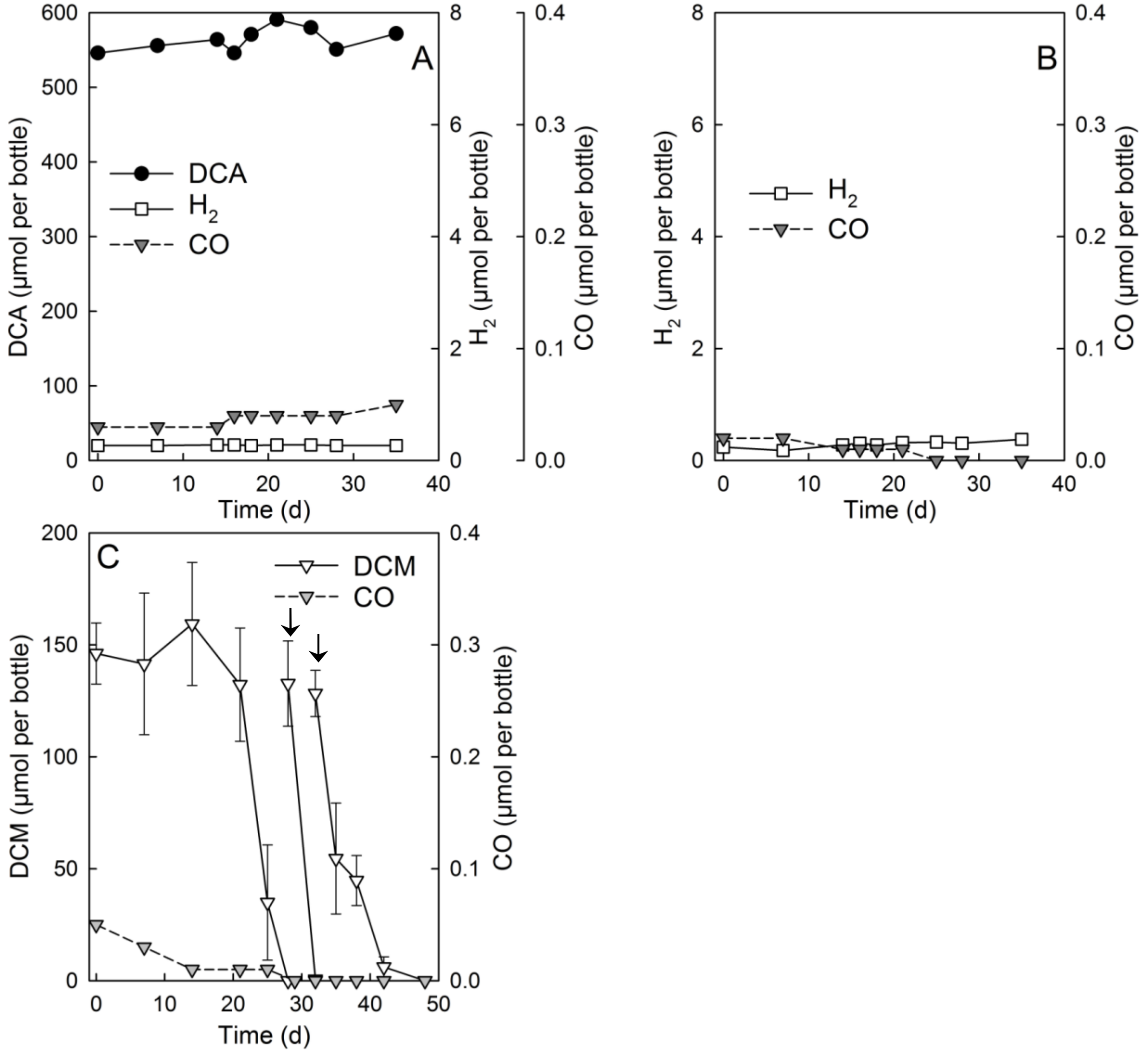

Fig. S1. Change of H_2 and CO concentrations in abiotic and biotic control incubations and CO concentrations in RM cultures during growth with DCM. Panel (A) shows H_2 and CO concentrations in an incubation with DCA but without inoculum (abiotic control). Panel (B) shows H_2 and CO concentrations in an incubation with inoculum but without DCA (biotic control). Neither H_2 nor CO formed in control incubations without DCA or without inoculum, demonstrating that the transient formation of H_2 and CO was dependent on the presence of active cells and DCA. Panel (C) shows CO concentrations in RM cultures during growth with DCM. In contrast to DCA-grown cultures shown in **Fig. 2**, CO formation was not observed during DCM catabolism. A small amount of CO (approximately 0.05 µmol) was present at the

start, which was completely consumed during the incubation. The arrows indicate two additional feedings with DCM. The data in panel (C) represent the averages of triplicate incubations and the error bars represent the standard deviations. Error bars smaller than the symbols are not shown. For ease of comparison, the Y-axis scales match those used in **Fig. 2**.
