# Supplementary material for: Anaerobic Microbial Metabolism of Dichloroacetate
Source: mBio. 2021 Apr 27;12(2):e00537-21. doi: 10.1128/mBio.00537-21 (PMC8092247; doi:10.1128/mBio.00537-21)
Supplement: FIG S2 [file mBio.00537-21-sf002.pdf]

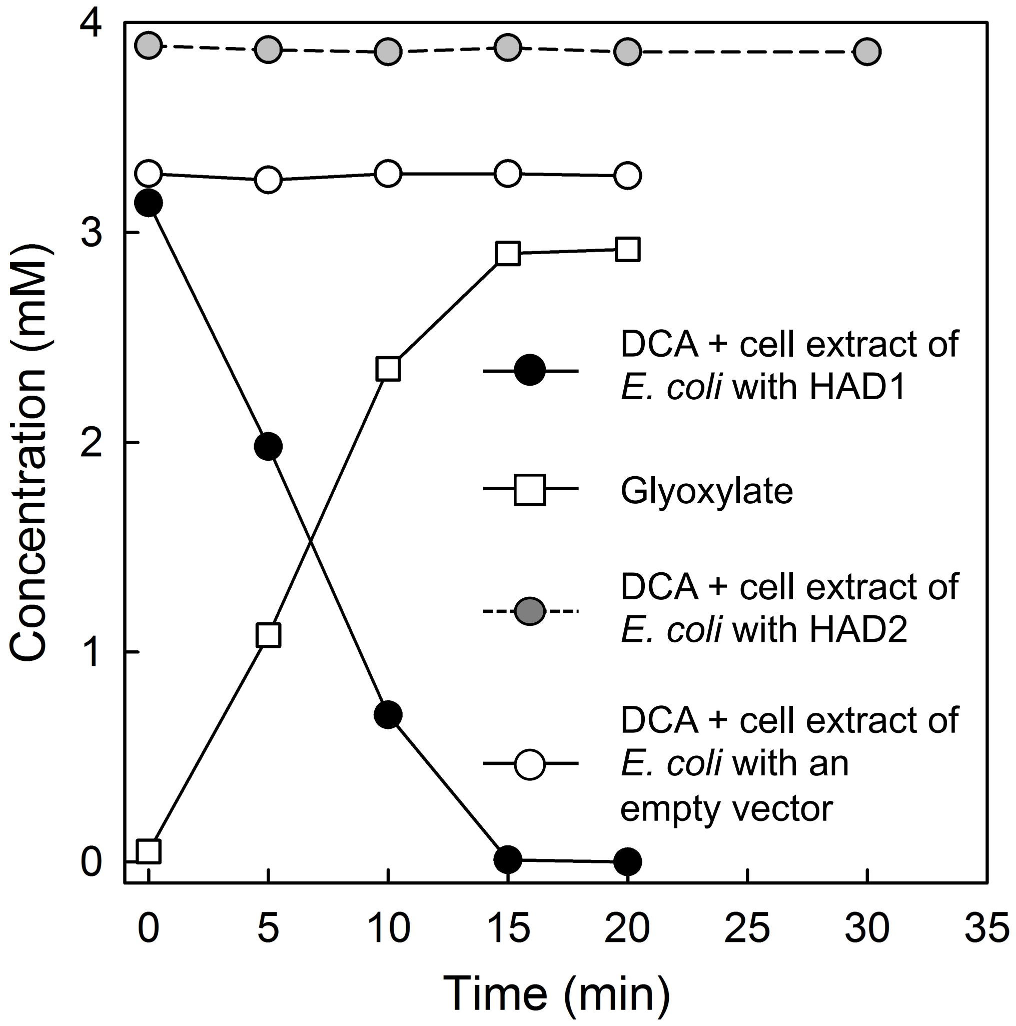

Fig. S2. Conversion of DCA to glyoxylate in cell-free extracts of *E. coli* transformants expressing the '*Ca.* Dichloromethanomonas elyunquensis' strain RM *had1* or *had2* gene. Cell-free extracts of the genetically modified *E. coli* strain FEL146 that expressed the strain RM *had1* gene (prokka_14346) stoichiometrically converted DCA (black circles) to glyoxylate (open squares). No DCA conversion occurred in assays with cell-free extracts of *E. coli* strain FEL145 (gray circles) expressing the *had2* gene (prokka_14344) and of *E. coli* strain FEL018 (open circles) carrying the empty vector.
