# Supplementary material for: Anaerobic Microbial Metabolism of Dichloroacetate
Source: mBio. 2021 Apr 27;12(2):e00537-21. doi: 10.1128/mBio.00537-21 (PMC8092247; doi:10.1128/mBio.00537-21)
Supplement: FIG S3 [file mBio.00537-21-sf003.pdf]

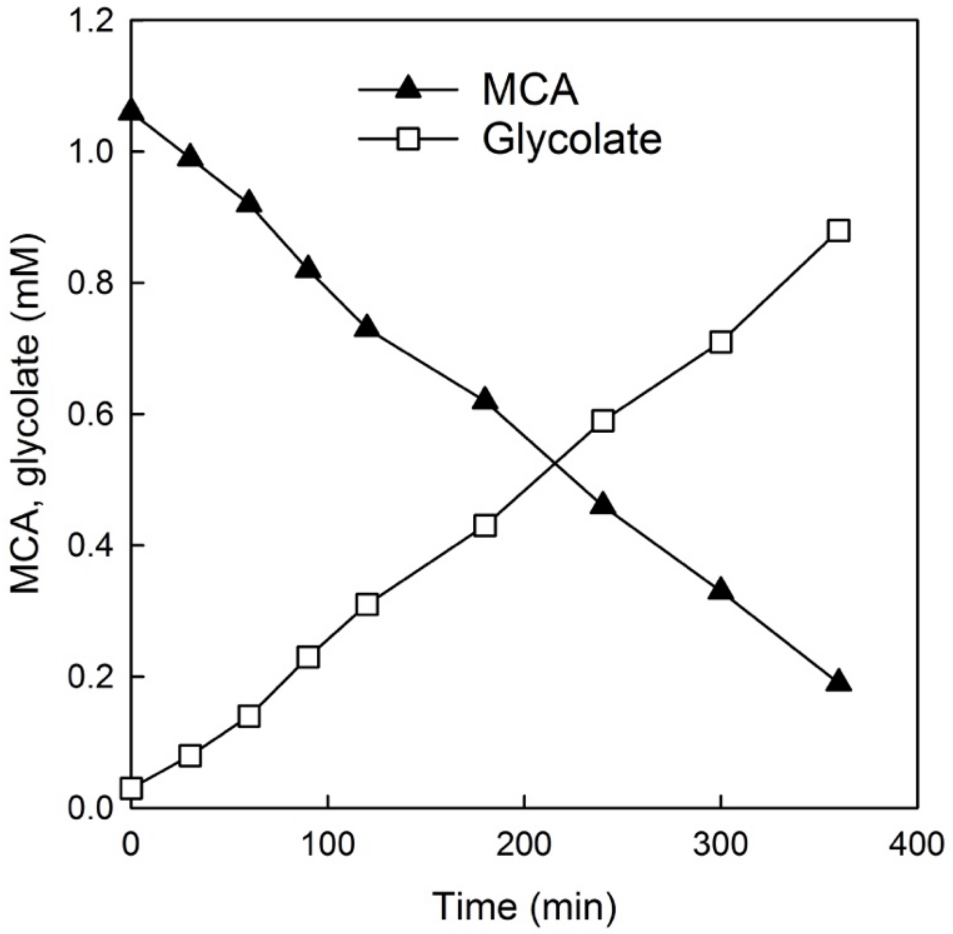

Fig. S3. *In vitro* conversion of monochloroacetate (MCA) to glycolate by the purified HAD1 protein. The enzyme assay was performed in 100 mM Tris/HCl buffer (pH 8.0) with an initial MCA concentration of approximately 1 mM. The total assay volume was 1 mL and 30 µg of purified HAD1 protein was added to start the reaction. MCA conversion to glycolate occurred at a rate of 1.3 nkat mg^{-1} protein, nearly 70-fold lower than the rate of DCA conversion to glyoxylate under identical experimental conditions.
