# Supplementary material for: Anaerobic Microbial Metabolism of Dichloroacetate
Source: mBio. 2021 Apr 27;12(2):e00537-21. doi: 10.1128/mBio.00537-21 (PMC8092247; doi:10.1128/mBio.00537-21)
Supplement: FIG S4 [file mBio.00537-21-sf004.pdf]

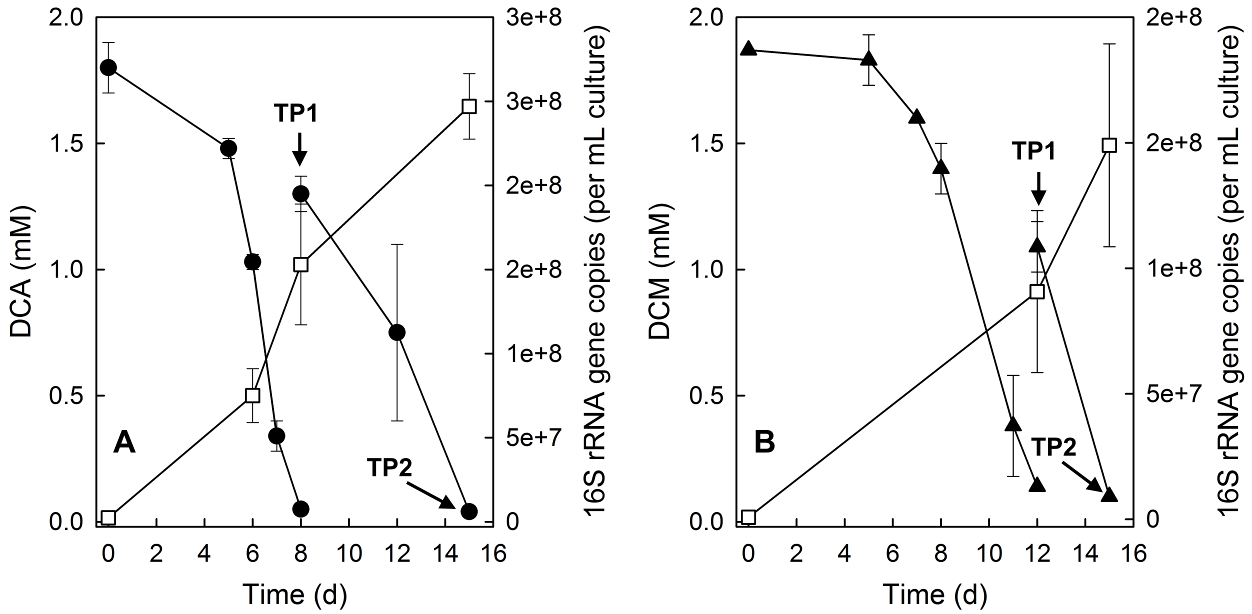

Fig. S4. Growth of ‘*Ca.* Dichloromethanomonas elyunquensis’ strain RM in mixed culture RM with DCA (A, closed circles) or DCM (B, closed triangles) as revealed with a specific 16S rRNA gene-targeted qPCR assay (open squares). The cultures received additional feedings of DCA and DCM following depletion of the initial amount of substrate. Sampling time points (i.e., TP1 and TP2) for comparative proteome analysis are indicated by solid arrows. The data represent the averages of triplicate incubations and the error bars represent the standard deviations. Error bars smaller than the symbols are not shown.
