# Supplementary material for: Anaerobic Microbial Metabolism of Dichloroacetate
Source: mBio. 2021 Apr 27;12(2):e00537-21. doi: 10.1128/mBio.00537-21 (PMC8092247; doi:10.1128/mBio.00537-21)
Supplement: FIG S5 [file mBio.00537-21-sf005.pdf]

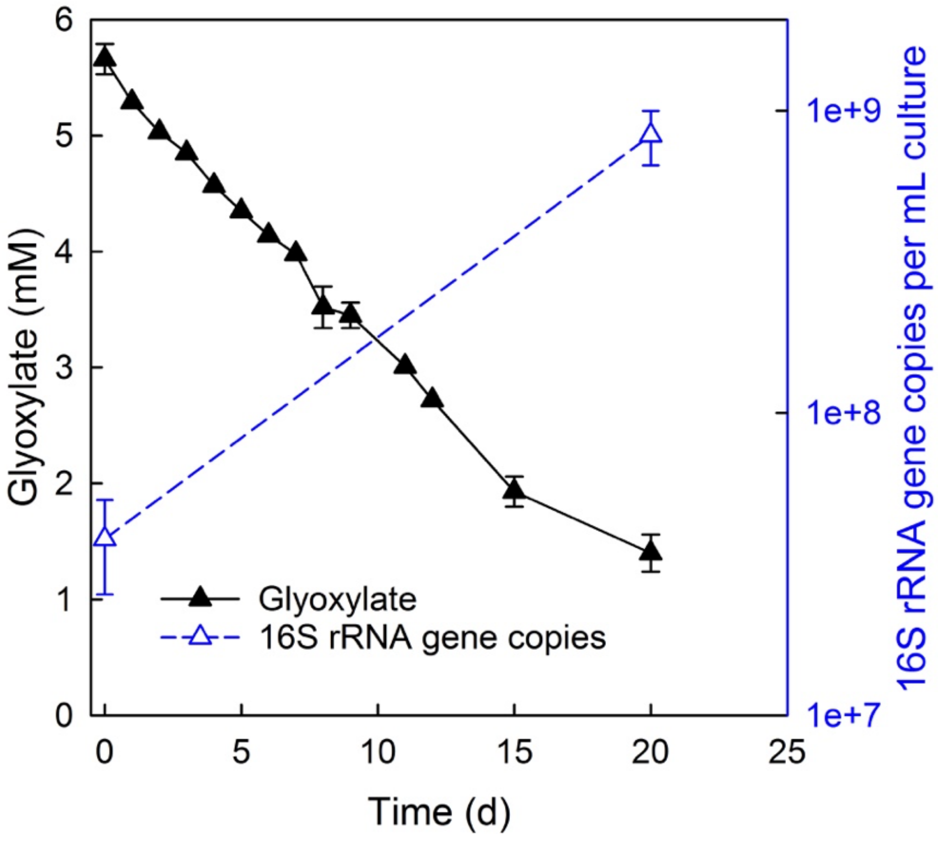

Fig. S5. Growth of '*Ca.* Dichloromethanomonas elyunquensis' strain RM in mixed culture RM with glyoxylate as the sole electron donor as determined by 16S rRNA gene-targeted qPCR. Bottles with defined mineral salt medium amended with glyoxylate as the sole energy source were inoculated (3%, v/v) from a DCA-grown culture. Glyoxylate was degraded and the '*Ca.* Dichloromethanomonas elyunquensis' 16S rRNA gene copy numbers increased during the incubation, indicating '*Ca.* Dichloromethanomonas elyunquensis' utilized glyoxylate as a growth substrate. The data represent the averages of triplicate incubations and the error bars represent the standard deviations. Error bars smaller than the symbols are not shown.
