# Supplementary material for: Anaerobic Microbial Metabolism of Dichloroacetate
Source: mBio. 2021 Apr 27;12(2):e00537-21. doi: 10.1128/mBio.00537-21 (PMC8092247; doi:10.1128/mBio.00537-21)
Supplement: FIG S6 [file mBio.00537-21-sf006.pdf]

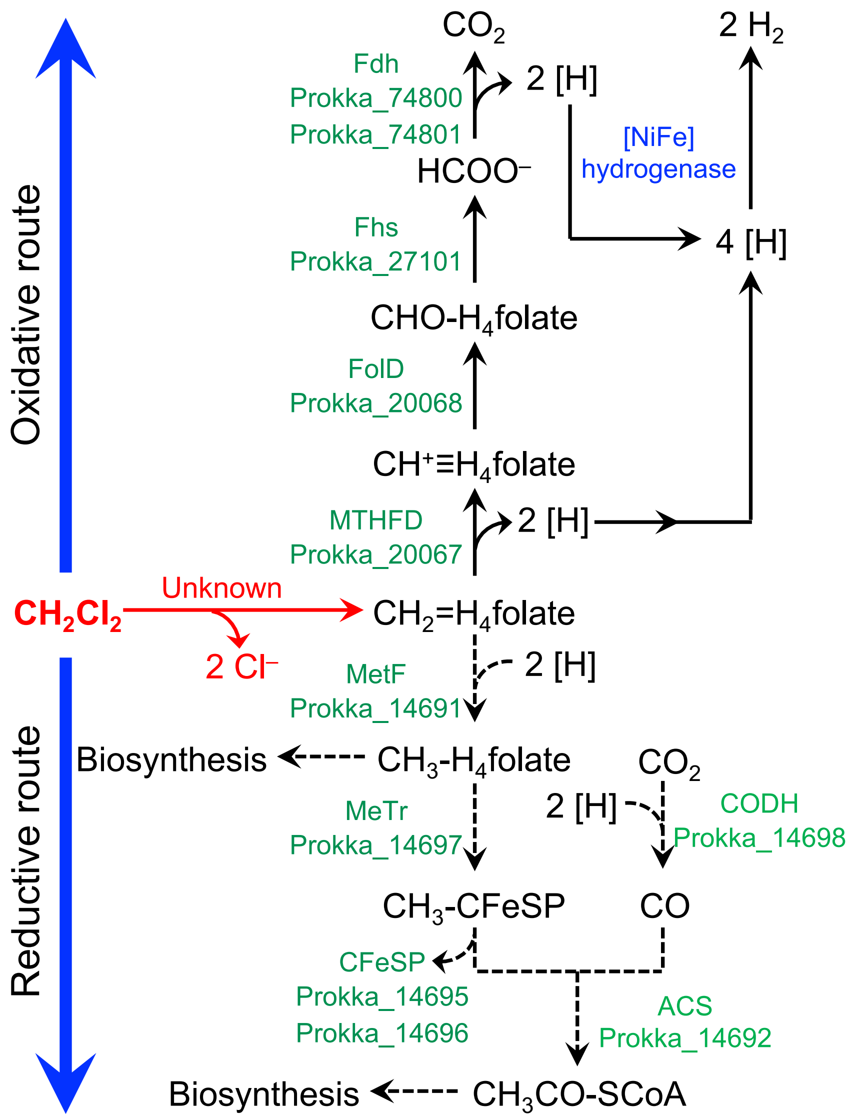

Fig. S6. Postulated anaerobic DCM catabolic pathway in '*Ca*. Dichloromethanomonas elyunquensis' strain RM. DCM is metabolized via the Wood–Ljungdahl pathway (WLP). DCM is initially dechlorinated and converted to methylene-tetrahydrofolate ($CH_2=H_4$folate) by a yet unknown enzyme. $CH_2=H_4$folate is completely mineralized to CO_2 and H_2 via the reversed WLP (oxidative direction, solid lines). A small part of $CH_2=H_4$folate is assimilated and further reduced via the WLP (dashed lines) to generate intermediates (e.g., CH_3-H_4folate and acetyl-CoA) for anabolic reactions. The complete WLP is encoded on the genome of strain RM and proteomics detected all of the WLP proteins. WLP proteins were expressed in DCA- and DCM-grown cells with higher abundances of most WLP proteins observed in cultures grown with DCM (see **Fig. 6**).
