# Supplementary material for: Anaerobic Microbial Metabolism of Dichloroacetate
Source: mBio. 2021 Apr 27;12(2):e00537-21. doi: 10.1128/mBio.00537-21 (PMC8092247; doi:10.1128/mBio.00537-21)
Supplement: FIG S7 [file mBio.00537-21-sf007.pdf]

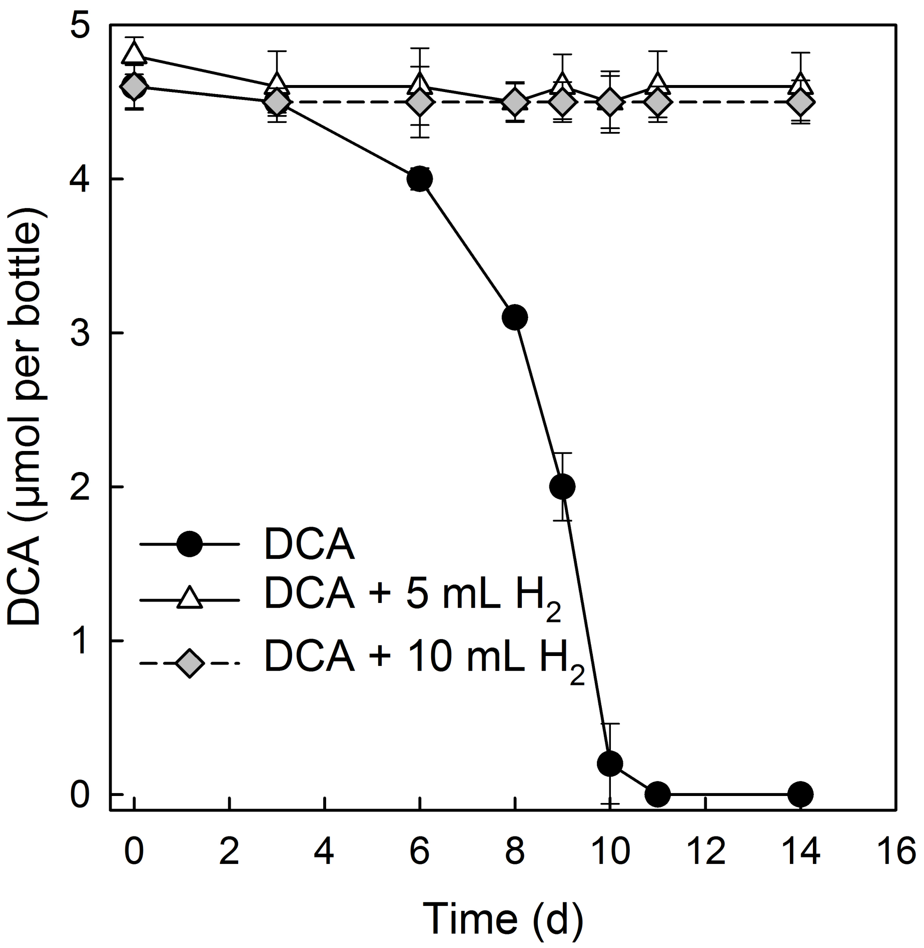

Fig. S7. Inhibitory effect of H_2 on DCA degradation in culture RM. The solid black circles show DCA degradation in culture RM without exogenous H_2 additions. Exogenously amended H_2 (5 mL, open triangles; 10 mL, gray diamonds) prevented DCA degradation. Experiments were performed in 160-mL serum bottles with 100 mL of anoxic mineral salt medium and 60 mL of headspace. The data represent the averages of triplicate incubations and the error bars represent the standard deviations. Error bars smaller than the symbols are not shown.
